# Neuroprotective Mechanisms of Hyperbaric Oxygen Therapy in Cerebral Ischemia-Hypoxia Injury Following Cardiopulmonary Resuscitation

**DOI:** 10.7150/ijms.123862

**Published:** 2026-01-14

**Authors:** Yifan Huang, Xiaopeng Liu, Xiaozhan Yang, Sisen Zhang

**Affiliations:** 1 Fifth Clinical Medical College, Henan University of Chinese Medicine, 39 Hongqi Road, Zhengzhou, Henan 450002, China.; 2 Emergency Medicine Department, Henan University of Chinese Medicine People's Hospital/ Zhengzhou People's Hospital, 33 Huanghe Road, Zhengzhou, Henan 450003, China.; 3 The Heart-lung-brain Resuscitation Engineering Technology Research Center of Henan Province, Zhengzhou, Henan 450003, China.

**Keywords:** hyperbaric oxygen therapy, cardiopulmonary resuscitation, hypoxic-ischemic brain injury, ferroptosis

## Abstract

Despite significant advancements in cardiopulmonary resuscitation (CPR) techniques, the global burden of sudden cardiac death remains high, with post-CPR survival rates persistently below 8%. Hypoxic-ischemic brain injury (HIBI) is the predominant cause of mortality, accounting for 68% of fatalities following resuscitation. Hyperbaric oxygen (HBO) therapy, which enhances oxygen dissolution in plasma, has demonstrated efficacy in focal cerebral ischemia conditions such as stroke. However, its potential in addressing global cerebral ischemia following CPR—a condition pathophysiologically distinct due to the absence of a salvageable ischemic penumbra and characterized by pan-cerebral energy failure—remains insufficiently explored. This review synthesizes emerging evidence from both focal and global ischemia models, highlighting the role of HBO in modulating key injury mechanisms common to both conditions, including oxidative stress, neuroinflammation, and ferroptosis. By integrating findings on HBO-induced upregulation of endogenous antioxidants, suppression of pro-inflammatory cytokines, and stabilization of mitochondrial function, we propose a combined therapeutic strategy that incorporates HBO with advanced CPR techniques and adjunctive therapies to mitigate HIBI.

## 1. Introduction

Cardiopulmonary resuscitation (CPR) is a critical life-saving intervention for patients experiencing early cardiac arrest (CA), as it facilitates the rapid restoration of respiratory and circulatory functions^1^. Despite its critical role, global outcomes remain poor, with sudden cardiac death claiming over 3 million lives annually and post-CPR survival rates below 8%. In China, where approximately 540,000 cases of cardiac arrest occur each year, survival rates are particularly alarming at less than 1%.

Hypoxic-ischemic brain injury (HIBI) is the principal determinant of poor prognosis after cardiac arrest, resulting from global cerebral ischemia during circulatory collapse and subsequent reperfusion injury upon flow restoration^2^. The neuronal damage in HIBI involves a spectrum of regulated cell death pathways. Apoptosis is a programmed, genetically controlled process crucial for eliminating damaged cells under physiological conditions, characterized by cell shrinkage and nuclear condensation^3^. However, under ischemic conditions, this process becomes dysregulated due to severe metabolic stress and mitochondrial dysfunction. The loss of metabolic homeostasis triggers sustained apoptotic signaling, which escalates uncontrollably and becomes a leading factor contributing to neuronal death, thereby exacerbating brain injury^4^, ^5^. This pathological shift underscores the critical role of uncontrolled apoptosis in HIBI pathogenesis.

Notably, more recently identified forms of regulated necrosis, such as ferroptosis—an iron-dependent pathway driven by lethal lipid peroxide accumulation—exhibit distinct mechanisms and contribute significantly to HIBI pathology. Furthermore, HIBI progression is exacerbated by dysfunction of the brain's protective barriers. The blood-brain barrier (BBB), formed by brain endothelial cells, regulates solute passage between blood and brain parenchyma, while the blood-cerebrospinal fluid barrier (BCSFB) at the choroid plexus controls exchange with cerebrospinal fluid; both are compromised during ischemia, facilitating neuronal injury^6^, ^7^.

Hyperbaric oxygen (HBO) therapy has emerged as a promising neuroprotective intervention. By delivering 100% oxygen at increased atmospheric pressures (typically 2-3 ATA), HBO dramatically enhances plasma oxygen solubility, elevating tissue oxygenation even in poorly perfused areas^8^. This mechanism supports cellular respiration and ATP production in ischemic tissues while simultaneously mitigating multiple injury pathways. Clinical applications already extend to various ischemic conditions, including stroke, traumatic brain injury, and carbon monoxide poisoning^9^.

**Figure 1:**
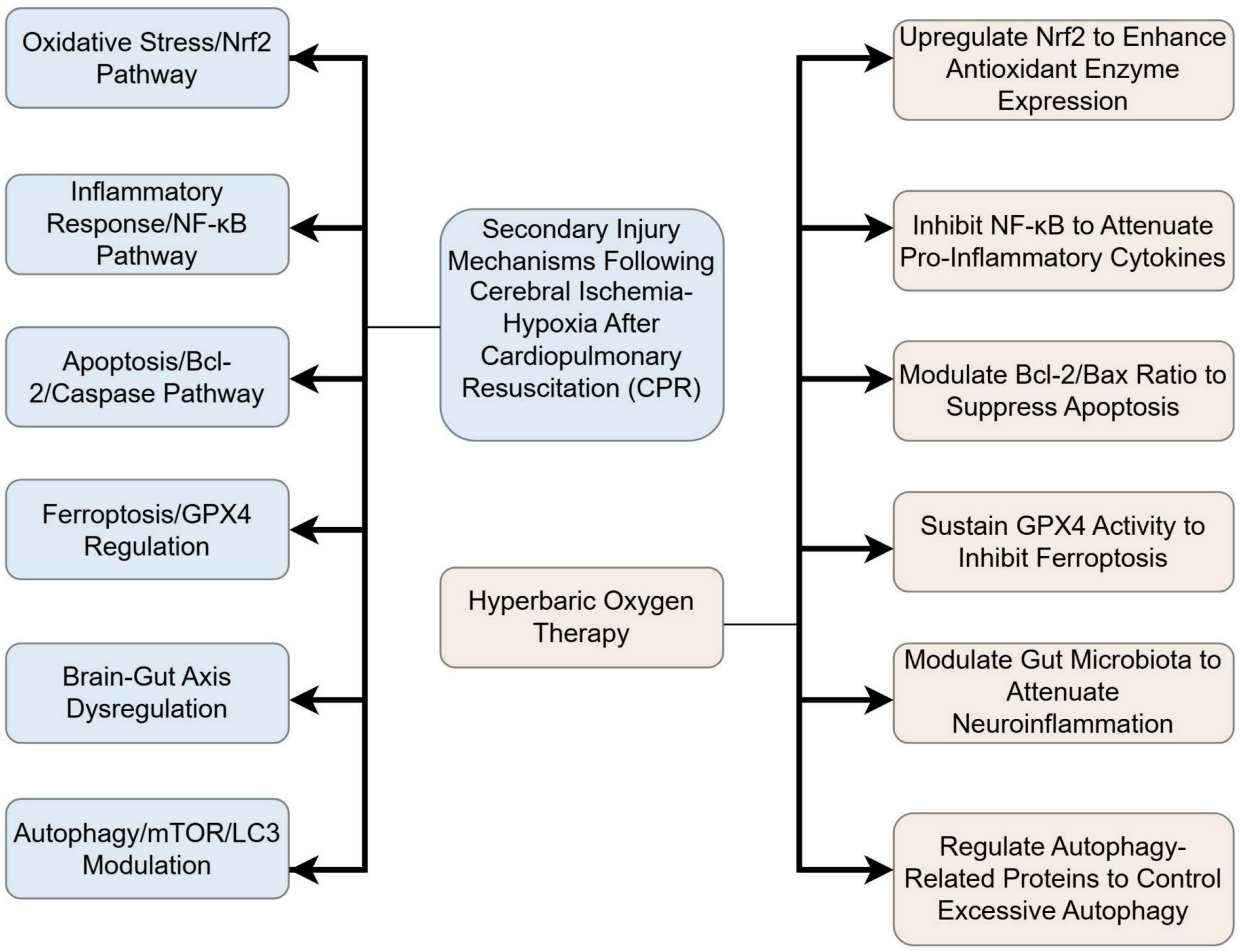
** Integrated Flowchart of HBO Neuroprotective Mechanisms.** ​​This diagram synthesizes key pathways, including anti-inflammatory, antioxidant, and anti-ferroptosis mechanisms.

It is important to note that cardiac arrest (CA) and the subsequent restoration of spontaneous circulation initiate a systemic ischemia-reperfusion process^10^-^12^. A pivotal characteristic of CA-induced cerebral injury is global ischemia, resulting from the abrupt cessation of systemic blood flow. This contrasts fundamentally with the focal ischemia typical of stroke, primarily due to the absence of a classical ischemic penumbra—a salvageable tissue region enabled by collateral circulation that is a target for revascularization therapies in stroke. The simultaneous cessation of global cerebral perfusion during CA precludes such collateral compensation^13^, ^14^. The core injury mechanism in CA-induced hypoxic-ischemic brain injury (HIBI) is pan-cerebral energy failure. This failure triggers rapid ATP depletion, leading to a cascade of events including cytotoxic edema, profound calcium overload, and severe mitochondrial dysfunction. This pathophysiology is distinct from stroke, where the injury is spatially heterogeneous and centered on a vascular occlusion site. Despite these macro-level pathological differences, significant convergence occurs at the molecular and cellular level. Both global ischemia post-CA and focal ischemia in stroke share key injury pathways, including excitotoxicity (e.g., NMDA receptor-mediated glutamate surge), oxidative stress from reactive oxygen species generation, and pronounced neuroinflammation involving microglial activation and cytokine release^15^. These initial insults collectively trigger downstream programmed apoptosis, which is characterized by mitochondrial dysfunction and caspase activation, ultimately leading to neuronal death^16^, ^17^. This mechanistic overlap provides a rationale for cautiously extrapolating insights from focal ischemia models to inform on HBO's potential mechanisms in post-CA global HIBI Figure 1.

## 2. Overview of the mechanisms of HIBI after CPR

**Figure 2:**
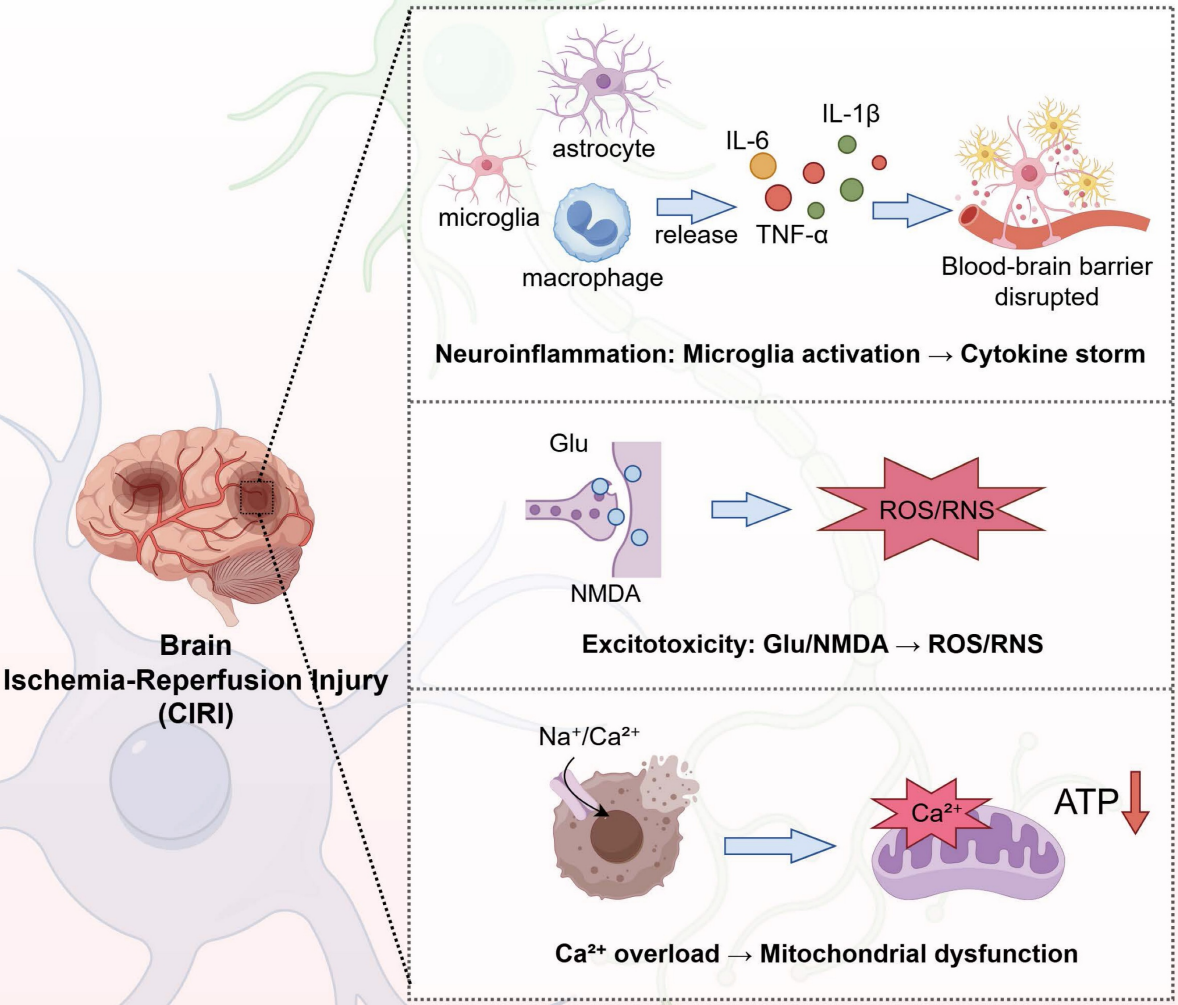
** Major pathophysiologic processes in cerebral ischemia-reperfusion injury.** IL: interleukin; TNF: tumor necrosis factor; ATP: adenosine triphosphate; ROS: reactive oxygen species; RNS: Reactive nitrogen species; Glu: glutamate; NMDA: N-methyl-d-aspartate.

After CPR, patients may experience a range of neurological complications, including shock, coma, seizures, neurocognitive dysfunction, memory impairment, and, in severe cases, brain death. Prolonged CA results in impaired cardiac muscle function and apnea, leading to a significant reduction in cerebral oxygenation and blood flow. Although CPR can restore respiratory and circulatory function, the brain may continue to suffer from localized ischemia and focal infarction. This persistent impairment in cerebral perfusion and oxygen delivery contributes to the development of HIBI. The main pathophysiological processes are shown in the following Figure 2.

### 2.1. Ischemia-reperfusion injury (IRI)

Cerebral hypoperfusion often occurs during the early stages after CPR, and cerebral vascular autoregulation is typically impaired within the first 24 hours. This dysfunction, combined with congestive blood flow during the recovery phase, exacerbates cerebral hypoxia and contributes to further neurological damage. In the later stages after CPR, as cerebral blood flow is restored, tissues may undergo IRI, which can lead to delayed and progressive neurological impairment^18^. However, the pathological mechanisms underlying cerebral ischemia reperfusion injury (CIRI) after CPR are very complex and involve calcium overload, mitochondrial dysfunction, inflammatory responses, apoptosis, the accumulation of oxygen free radicals, and the excessive release of excitatory amino acids^19^.

During cerebral ischemia-reperfusion injury (CIRI), the interruption of oxygen and glucose delivery triggers a critical shift from aerobic metabolism to inefficient anaerobic glycolysis, culminating in ATP depletion and failure of ionic homeostasis^20^. This energy crisis impairs Na⁺/K⁺-ATPase and Ca²⁺-ATPase function, leading to intracellular accumulation of Na⁺ and Ca²⁺. The overactivation of N-methyl-D-aspartate (NMDA) receptors after ischemic insult initiates a pathological cascade driven primarily by excessive calcium ion (Ca²⁺) influx into neurons. This sustained Ca²⁺ entry disrupts cellular energy homeostasis by overwhelming ATP-dependent ion pumps, including the Na⁺/K⁺-ATPase and plasma membrane Ca²⁺-ATPases, which are critical for maintaining ionic balance. The resulting membrane depolarization facilitates additional Ca²⁺ influx through voltage-gated channels, further exacerbating intracellular Ca²⁺ loading. In response to cytosolic Ca²⁺ overload, mitochondria sequester calcium via the mitochondrial calcium uniporter (MCU). Under physiological conditions, this buffering mechanism is protective; however, under excitotoxic conditions, excessive MCU-mediated Ca²⁺ uptake induces mitochondrial permeability transition pore (mPTP) opening, uncouples oxidative phosphorylation, and elevates reactive oxygen species (ROS) production^21^, ^22^.

These alterations impair electron transport chain function, leading to severe ATP depletion. The ensuing energy deficit, combined with oxidative stress, perpetuates a self-sustaining cycle of metabolic dysfunction, ultimately driving neuronal death through necrotic and apoptotic pathways^23^.

Within the mitochondria, excessive Ca²⁺ disrupts the electron transport chain, impairing ATP synthesis and stimulating the generation of reactive oxygen species (ROS), thereby inducing profound mitochondrial dysfunction^24^. The ensuing oxidative stress further exacerbates the initial insult, creating a vicious cycle of metabolic failure and neuronal injury^25^.

Moreover, CIRI activates resident immune cells in the brain, including microglia and astrocytes, as well as infiltrating macrophages. These immune cells release a cascade of pro-inflammatory cytokines such as tumor necrosis factor-alpha (TNF-α), interleukin-6 (IL-6), and interleukin-1 beta (IL-1β), which contribute to mitochondrial damage, endothelial injury, and increased blood-brain barrier (BBB) permeability. The resulting breakdown of the BBB exacerbates brain tissue damage and worsens the neurological outcome^26^.

### 2.2. Apoptosis and necrosis

Several hours to days after CA, regions of the brain with high metabolic demands—such as the hippocampus, white matter centers, and basal ganglia—are among the first to sustain damage due to their critical reliance on adequate oxygen and blood supply. Histopathological examination of these regions typically reveals hallmark features of cellular injury, including mitochondrial and endoplasmic reticulum swelling, nuclear chromatin condensation, and evidence of both necrosis and apoptosis in neuronal cells. Apoptosis plays a central role in CIRI^27^ and is a major contributor to neuronal death. The B-cell lymphoma-2 (BCL-2) protein family, particularly the pro-apoptotic protein Bax and the anti-apoptotic protein BCL-2, are key regulators of the mitochondrial apoptotic pathway. Upon activation of apoptotic signaling, Bax interacts with BCL-2^28^, disrupting mitochondrial membrane integrity. This disruption facilitates the release of cytochrome c into the cytoplasm, triggering caspase^29^ activation and leading to apoptotic cell death.

These different factors interact with each other at multiple points in the development of HIBI, and together, they lead to neuronal cell damage, necrosis, or apoptosis in brain tissues. Notably, HIBI typically manifests in the later stages following CPR and results from a combination of primary ischemic insult and secondary injury processes. Therefore, effective treatment of HIBI after CPR requires not only the prompt restoration of cerebral perfusion but also the mitigation of ongoing neurological damage. Emerging evidence suggests that HBO therapy may offer neuroprotective benefits in this context. By increasing arterial oxygen partial pressure and enhancing oxygen solubility in plasma, HBO promotes cellular respiration and ATP synthesis in ischemic and hypoxic tissues. This mechanism helps to correct cerebral hypoxia and reduce the extent of brain injury^30^.

## 3. Mechanism underlying the protective effect of HBO on HIBI

**Figure 3:**
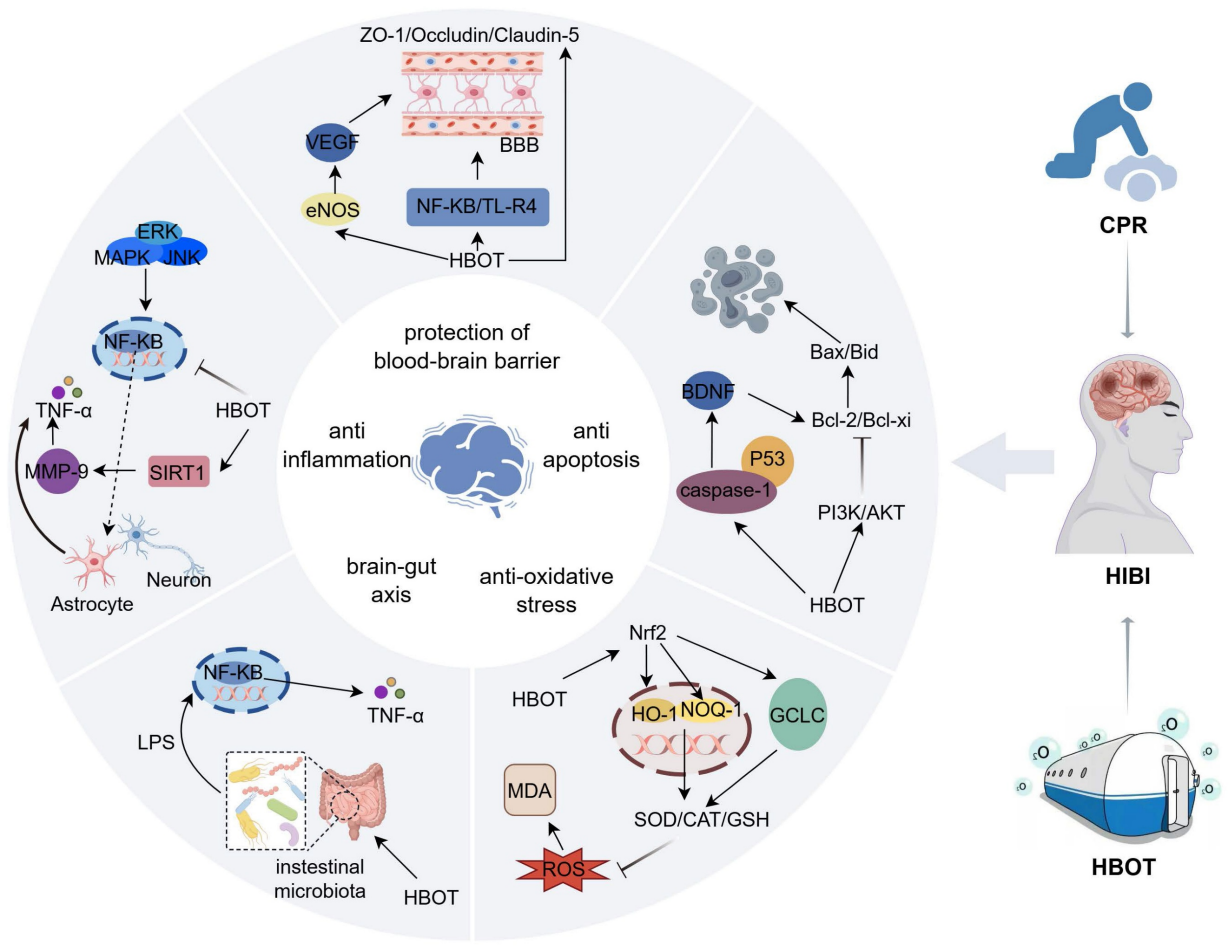
** Schematic showing the primary mechanisms of HBO's protective effect on HIBI.** It is described that HBO can play a protective role during cerebral ischemia and hypoxia by protecting the blood-brain barrier, inhibiting oxidative stress, attenuating the inflammatory response, inhibiting apoptosis, and regulating the brain-gut axis. Inhibition of inflammatory response and anti-oxidative stress are the main protective mechanisms of HBO against cerebral ischemia-hypoxia injury. Inflammatory response is one of the important factors inducing cerebral ischemia, which is prone to cause cerebrovascular circulatory disorders, triggering a series of cascade reactions such as apoptosis and oxidative stress, so inhibiting the inflammatory response improves neurological function injury. In the regulatory mechanism of HBO-mediated inflammatory response, the expression of upstream targets and inflammatory factors is regulated through the NF-κB core signaling pathway to inhibit the release of pro-inflammatory factors involved in the inflammatory response. In the anti-oxidative stress regulatory network, Nrf2 is the upstream pathway, which is involved in regulating the expression of various antioxidant enzymes such as CAT, SOD, NQO-1, HO-1, etc., and decreasing the intracellular reactive oxygen species and malondialdehyde content. However, the research on HBO in protecting the blood-brain barrier and regulating the cerebral-intestinal axis is insufficient. The brain-gut axis is the bridge between the nervous system and the intestine. After cardiopulmonary resuscitation, cerebral ischemia and hypoxia will rapidly trigger intestinal flora disorders, and intestinal flora dysregulation can further aggravate brain injury by regulating the immune system.

Hyperbaric oxygen therapy involves the administration of 100% oxygen in a pressurized environment, typically at two to three times the atmospheric pressure at sea level^31^. This treatment modality has been increasingly recognized for its ability to alleviate cerebral ischemic-hypoxic injury caused by various pathological Figure 3.

### 3.1. HBO preconditioning induces tissue ischemic tolerance

Hyperbaric oxygen (HBO) preconditioning involves the short-term application of HBO (e.g., 2.0-2.5 ATA) prior to an anticipated ischemic insult. This strategy aims to enhance intrinsic cellular defense mechanisms, thereby increasing tissue resilience^31^. The protective effects are mediated through the upregulation of key endogenous factors, including ​​hypoxia-inducible factor-1 alpha (HIF-1α)​​ and heat shock protein 70 (HSP70)^32^. The apparent discrepancy regarding HIF-1α regulation—wherein HBO preconditioning upregulates it, while post-injury HBO therapy may downregulate it—can be explained by the ​​temporal context and the distinct oxygen dynamics​​ involved. During preconditioning, the cyclic nature of HBO exposure (intermittent hyperoxia followed by a return to normoxia) creates a state of ​​controlled relative hypoxia​​ within the tissue Table 1. This transient relative hypoxia stabilizes HIF-1α, leading to its accumulation and the subsequent activation of adaptive genes such as erythropoietin (EPO)^33^, which promotes cell survival and angiogenesis. This process mimics the protective effects of ischemic preconditioning. Furthermore, the upregulation of HSP72 is a well-documented response to HBO preconditioning. HSP72 facilitates the stabilization and transcriptional activity of HIF-1α, creating a synergistic cytoprotective loop. Numerous animal studies have confirmed that this adaptive response significantly increases the threshold for neuronal damage following subsequent ischemic-hypoxic events.

### 3.2. Improvement of brain tissue oxygen supply and metabolic recovery

Under hyperbaric conditions, the physical solubility of oxygen in the blood increases approximately 17 to 20 times compared to that under normobaric atmospheric pressure. This substantial increase in dissolved oxygen significantly enhances the oxygen-carrying capacity of the blood, allowing it to meet the metabolic demands of the body more effectively^34^. Moreover, elevated oxygen levels facilitate the rapid diffusion of oxygen across the blood-cerebrospinal fluid barrier, enabling oxygen delivery to injured brain tissue. This process helps reduce mitochondrial dysfunction in ischemic regions, prevents a shift to anaerobic metabolism, and alleviates hypoxia in affected brain cells^35^. Although cardiac arrest induces global cerebral ischemia rather than focal ischemia, the restoration of oxygen supply and metabolic homeostasis represents a core component of mitigating neuronal injury. Research conducted in a middle cerebral artery occlusion (MCAO) model of focal ischemia demonstrated that both normobaric oxygen therapy (NBO; 100% O₂) and hyperbaric oxygen therapy (HBO; 3 ATA of 100% O₂ for 60 minutes) effectively attenuated tissue acidosis, as measured by umbelliferone fluorescence, and significantly improved energy metabolism in the ischemic regions. These findings suggest that by targeting this shared mechanism of correcting hypoxia and metabolic dysfunction, HBO may exert similar protective effects in the context of global cerebral ischemia following cardiac arrest, although further validation is required^36^, ^37^.

### 3.3. Inhibition of apoptosis and necrosis

Apoptosis typically begins several hours after the onset of cerebral ischemia and is primarily localized to the ischemic penumbra. This form of programmed cell death is largely regulated by mitochondrial pathways involving the Bcl-2 family of proteins and the cysteine-aspartate protease (caspase) family, both of which contribute to neuronal apoptosis in ischemic brain regions^38^. While cardiac arrest induces global ischemia, it shares neuronal apoptosis pathways with focal ischemia. A study demonstrated that HBO therapy (2.5 ATA, 2 hours) significantly suppressed caspase-3 activation in NeuN-positive neurons and reduced DNA fragmentation in the ischemic cortex of MCAO/R model rats, indicating that HBO confers neuroprotection by attenuating neuronal apoptosis^39^. Critically, the therapeutic efficacy of HBO may involve the modulation of the transcription factor p53, a central regulator of apoptosis activated under ischemic stress.​​ Research indicates that hypoxic conditions can induce a conformational change in p53^40^, rendering it transcriptionally inactive. Notably, re-oxygenation strategies, which share the core aim of HBO to alleviate tissue hypoxia, have been shown to ​​restore the wild-type conformation and transcriptional activity of p53​​. This reactivation promotes the expression of pro-apoptotic genes, thereby facilitating the elimination of damaged neurons. Consequently, HBO therapy may ameliorate cognitive deficits not only by directly modulating the Bcl-2/Bax balance and reducing caspase-3 activity but also potentially through this p53-dependent pathway, collectively promoting neuronal survival.

Additionally, the improvement in cognitive function observed following hyperbaric oxygen (HBO) therapy is a downstream effect of its core ability to provide broad neuroprotection and halt the progression of brain injury. This protective action is mediated by a network of interconnected mechanisms: it suppresses neuronal apoptosis by modulating key regulators like the Bax/Bcl-2 balance and reducing caspase-3 activity; it facilitates the clearance of pathogenic protein aggregates such as β-amyloid; and it attenuates cellular senescence by lowering the expression of markers including p16, p21, and p53^41^. In parallel, HBO therapy fosters an environment conducive to neural repair, exemplified by the upregulation of neurotrophic factors like brain-derived neurotrophic factor (BDNF) in the hippocampus. Consequently, the amelioration of cognitive deficits directly results from this coordinated mitigation of the initial brain injury^42^-^44^.

Furthermore, HBO has been found to activate the phosphatidylinositol 3-kinase (PI3K) /Akt/ mammalian target of the rapamycin (mTOR) signaling pathway. This activation increases the expression of PI3K, mTOR, and Bcl-2, as well as the ratio of phosphorylated Akt to total Akt, while concurrently downregulating Bax expression. These molecular changes result in reduced apoptosis in basilar artery endothelial cells and contribute to improved neurological outcomes^45^. Several studies^46^, ^47^ have confirmed that HBO plays a role in attenuating brain damage by inhibiting apoptosis and necrosis.

### 3.4. Anti-oxidative stress and mitochondrial protection

The major pathophysiological component of cerebral ischemic-hypoxic injury is the production of large amounts of free radicals, which further damage lipids, proteins, and deoxyribonucleic acid (DNA), thereby inducing neuronal cell death^48^. During ischemic-hypoxic events, large quantities of ROS—including superoxide (O₂⁻), hydrogen peroxide (H₂O₂), peroxynitrite (ONOO⁻), and hydroxyl radicals—are generated. These reactive species promote lipid peroxidation, disrupt cell membrane integrity, increase malondialdehyde (MDA) release, and cause DNA damage, collectively contributing to cell dysfunction and death^49^.

Hyperbaric oxygen (HBO) therapy mitigates reactive oxygen species (ROS) accumulation primarily by activating the nuclear factor erythroid 2-related factor 2 (Nrf2) signaling pathway Figure 3. Upon activation, Nrf2 translocates to the nucleus and coordinates the upregulation of a suite of cytoprotective genes^50^. Key downstream effectors include heme oxygenase-1 (HO-1), NAD(P)H quinone oxidoreductase 1 (NQO1), and the catalytic subunit of glutamate-cysteine ligase (GCLC), which is the rate-limiting enzyme in glutathione (GSH) synthesis. This transcriptional program enhances cellular antioxidant capacity through a dual mechanism: it boosts the activity of enzymes like superoxide dismutase (SOD) and facilitates the synthesis of crucial non-enzymatic antioxidants such as GSH^51^, ^52^. Consequently, this coordinated response effectively reduces biomarkers of oxidative damage like malondialdehyde (MDA), underpinning the neuroprotective effects of HBO^53^.

In a study utilizing a specific hyperbaric oxygen (HBO) regimen (2.5 ATA, 1-hour sessions, twice daily for 2 consecutive days), treatment was found to activate the Nrf2 signaling pathway in neonatal rat brain tissue subjected to hypoxic-ischemic insult. This activation led to the upregulation of key downstream antioxidant proteins, including heme oxygenase-1 (HO-1) and glutathione S-transferase (GST). The consequent enhancement of the cellular antioxidant defense system effectively alleviated oxidative stress, which was associated with a significant reduction in cerebral infarct volume and neuronal apoptosis, thereby contributing to improved neurological function^54^. Therefore, the antioxidant and mitochondrial protective effects of HBO therapy, demonstrated in focal ischemia models via mechanisms such as Nrf2 pathway activation, may also hold significant relevance for global cerebral ischemia following cardiac arrest and cardiopulmonary resuscitation (CPR), given the central role of oxidative stress and bioenergetic failure in both conditions. This mechanistic synergy suggests that HBO could potentially ameliorate CPR-related brain injury by countering the pervasive oxidative damage and mitochondrial dysfunction characteristic of post-cardiac arrest syndrome^55^.

### 3.5. Inhibition of the inflammatory response

Several studies^33^have demonstrated that HBO improves neurological outcomes^56^ in animal models of brain injury by modulating the activation of microglia and astrocytes through multiple signaling pathways^57^. This modulation leads to a reduction in the release of pro-inflammatory cytokines such as IL-6, IL-1β, and TNF-α, mitigates damage to the BBB, and promotes both angiogenesis and neurogenesis^58^. It has been found that the inflammatory response is mediated by many signaling pathways, among which, nuclear factor-κB (NF-κB) is a key factor in the inflammatory response signaling pathway^59^. Upon activation during ischemic events, NF-κB facilitates the polarization of microglia toward the pro-inflammatory M1 phenotype, thereby enhancing the production of cytokines such as IL-1β and TNF-α^60^, as well as reactive oxygen species. This cascade amplifies the inflammatory response and exacerbates neuronal injury^61^. HBO therapy has been shown to regulate proteins upstream of NF-κB, inhibit its activation, and consequently suppress downstream inflammatory responses. Liu et al.^57^. proposed an alternative mechanism underlying the neuroprotective effects of HBO therapy, demonstrating through *in vitro* cell-based experiments that HBO attenuates brain injury-induced inflammatory responses by significantly downregulating the expression of the key chemokine CXCL1 and its receptor CXCR2. This effect is achieved through inhibition of the lipopolysaccharide (LPS)-induced NF-κB/mitogen-activated protein kinase (MAPK)-mediated CXCR2/CXCL1 signaling pathway. CXCL1 is predominantly expressed in astrocytes, whereas its receptor, CXCR2, is mainly found in neurons. In the context of traumatic brain injury, HBO has been shown to reduce neuronal apoptosis and mitigate secondary injury via this same pathway^62^. Additionally, HBO modulates neuroinflammation by downregulating the expression of C-C chemokine ligand 2 (CCL2), its receptor CCL2, and phosphorylated p38 through the p38-MAPK-CCL2 signaling axis^63^. Xue *et al.* further reported that HBO therapy at 2.5 ATA was more effective than treatment at 1.5 ATA in enhancing memory performance and reducing inflammatory responses in rats, suggesting a pressure-dependent therapeutic effect^64^. Previous studies have shown that silencing regulator protein 1 (sirtuin 1, SIRT1), a NAD^+^-dependent deacetylase, plays a crucial role in inhibiting inflammatory responses^65^, attenuating cerebral ischemia-reperfusion injury, and promoting neurological recovery^66^. Experimental knockdown of SIRT1 has been found to induce neuroinflammatory damage in cells. In contrast, HBO therapy activates SIRT1 expression, leading to a reduction in the release of inflammatory factors such as TNF-α, IL-1β, and IL-6. Additionally, HBO ameliorates ischemic-hypoxic brain injury by regulating SIRT1-induced deacetylation of High mobility group Box 1 (HMGB1), which inhibits matrix metalloproteinase-9 (MMP-9)^67^. The study conducted in a spinal cord injury (SCI) model demonstrated that hyperbaric oxygen therapy administered at 2-3 ATA not only upregulates the plasma anti-inflammatory cytokine interleukin-4 but also enhances the expression of SIRT1 and the mitochondrial marker voltage-dependent anion-selective channel (VDAC). This upregulation promotes mitochondrial biogenesis, reduces apoptotic signaling, and inhibits inflammatory cascade responses, highlighting a mechanism through which HBO attenuates inflammation^68^, By modulating these conserved cascades—notably via SIRT1 activation and regulation of apoptosis—HBO therapy contributes to improved functional recovery following central nervous system injuries, suggesting its potential to mitigate post-cardiac arrest encephalopathy by targeting shared inflammatory and apoptotic pathways in global cerebral ischemia^69^, ^70^.

### 3.6. Inhibition of ferroptosis

Ferroptosis is a newly recognized mode of cell death, characterized primarily by the activation of iron-dependent lipid peroxidation^71^, leading to the accumulation of peroxidation products^72^. This process is typically accompanied by the downregulation of the antioxidant functions of GSH and glutathione peroxidase 4 (GPX4). Nrf2, a key regulator of oxidative stress, acts as a negative regulator of ferroptosis by maintaining intracellular redox homeostasis. It achieves this by mediating the expression of antioxidant enzyme genes, decreasing intracellular Fe²⁺ levels, inhibiting ROS production, and thus preventing ferroptotic cell death.

Chen *et al.*^73^ demonstrated that hyperbaric oxygen (HBO) therapy ameliorates cerebral ischemia-reperfusion injury (CIRI) by suppressing ferroptosis. In their rat model, pathological alterations indicative of ferroptosis—including mitochondrial cristae dissolution, vacuolization, elevated ferritin and malondialdehyde (MDA), and reduced glutathione (GSH) observed in untreated CIRI controls—were significantly reversed following a 30-day regimen of 2.5 ATA HBO, underscoring its protective role. This aligns with evidence that HBO modulates key ferroptosis regulators, such as GPX4 and SLC7A11, to attenuate iron-dependent lipid peroxidation^74^. Therefore, HBO-mediated ferroptosis inhibition represents a pivotal neuroprotective mechanism, particularly in mitigating global cerebral injury following cardiac arrest and cardiopulmonary resuscitation, by preserving neuronal viability under ischemic stress.

### 3.7. Regulation of autophagy activation

Autophagy plays a context-dependent role in cerebral ischemia, exhibiting a dual nature that is critical for therapeutic targeting. While it's early, controlled activation promotes neuroprotection by clearing damaged organelles and misfolded proteins; excessive or prolonged autophagic activity can culminate in programmed cell death, thereby exacerbating ischemic brain injury^75^-^77^. The mechanistic target of rapamycin (mTOR) is a central regulator of this process, serving as a key inhibitory checkpoint of autophagy^78^. HBO therapy appears to fine-tune this delicate balance by modulating the expression of critical autophagy-related proteins, including mTOR, phosphorylated mTOR (p-mTOR), and the lipidated form of microtubule-associated protein light chain 3 (LC3B-II). Furthermore, by downregulating the upstream hypoxia-inducible factor-1α (HIF-1α), HBO may indirectly influence the autophagic cascade, contributing to its ameliorative effects on cerebral ischemic-hypoxic injury. Consequently, the precise regulation of the extent and timing of autophagy represents a promising yet complex mechanism underpinning HBO's therapeutic potential^42^, ^75^, ^79^.

Therefore, HBO's capacity to fine-tune autophagic activity—promoting its protective role in cellular clearance while curtailing its detrimental progression to programmed cell death—highlights its potential not only in focal ischemia but also in addressing global cerebral injury following cardiac arrest and CPR, where dysregulated autophagy significantly contributes to neuronal damage. This nuanced regulation, mediated through key pathways such as mTOR signaling and HIF-1α modulation, warrants further investigation in physiologically relevant models of post-cardiac arrest syndrome to fully elucidate its translational promise^80^.

### 3.8. Regulation of the brain-gut axis

The brain-gut axis is a bidirectional communication pathway that connects the central nervous system with the gastrointestinal tract. Alterations^81^ in the intestinal microbiota can lead to abnormal immune function in the small intestine, which mediates inflammation following brain injury and plays a crucial role in triggering brain-gut axis disorders^82^.A recent study discovered that pro-inflammatory the triggering receptor expressed on myeloid cell 1 (TREM1) signaling conveyed by gut-derived macrophages is transported to the brain and plays a significant role in the pathophysiology of secondary brain injury following CA/CPR^83^.

In 2024, Nyam *et al.* demonstrated for the first time that HBO therapy can influence the composition of gut microbiota after traumatic brain injury. They applied 2.0 ATA for 60 minutes at three distinct time points: immediately after brain injury, 24 hours later, and 48 hours later. Measurements taken three days post-injury revealed a reduction in the volume of brain damage and downregulation of inflammatory factors in rats. Since the intestinal flora consists predominantly of anaerobic bacteria (approximately 90%), the study found that five genus-level bacteria and two species-level bacteria of the core intestinal microbiota decreased 72 hours after brain injury following HBO treatment. This suggests that an increase in tissue oxygenation can directly affect microbial composition, reducing the prevalence of anaerobic bacteria^84^.

In conclusion, while direct evidence is currently lacking, preclinical insights suggest a compelling possibility that HBO therapy can reduce cerebral injury following cardiac arrest and CPR by modulating the brain-gut axis. This potential mechanism could involve the restoration of microbiota balance and suppression of TREM1-mediated neuroinflammation, but awaits direct validation in models of global cerebral ischemia.

### 3.9. Regulation of blood-brain barrier permeability, improvement of collateral circulation establishment, and neuronal cell regeneration

HBO therapy significantly attenuates brain edema in rats with early brain injury (EBI) following subarachnoid hemorrhage (SAH). It also alleviates BBB permeability and ultrastructural damage by inhibiting the Toll-like receptor 4 (TLR4)/NF-κB signaling pathway, thereby preventing the initiation of the innate immune response and inflammation-related gene transcription. Hao *et al.* demonstrated that HBO upregulated the expression of connexins, such as occludin and zonula occludens-1 (ZO-1), in hypoxic cells, suggesting that HBO helps maintain the integrity and permeability of the BBB^85^. The Wnt/β-catenin (β-catenin) signaling pathway plays a critical role in the formation and maintenance of the BBB, with its activation being essential for preserving BBB integrity after cerebral ischemia^86^.

Endothelial nitric oxide synthase (eNOS) is a key factor in endothelium-dependent vasodilation, responsible for the synthesis of nitric oxide (NO), which promotes vascular smooth muscle relaxation and increases cerebral blood flow. It has been shown that HBO therapy can enhance nitric oxide levels by activating nitric oxide synthase, upregulate eNOS expression, and facilitate the recovery of injured blood vessels after ischemic events^87^. Furthermore, animal studies have revealed that HBO can promote the expression of endothelial growth factors in the vasculature of rats with acute cerebral infarction, thereby aiding in the establishment of collateral circulation, neovascularization, and improving hemodynamic stability^88^. Other studies^45^, ^89^ have found that HBO treatment promotes the expression of nerve growth factor in brain cells, thereby supporting the recovery of neurological function in rats with craniocerebral injuries. In summary, the beneficial effects of HBO on BBB integrity, collateral circulation, and angiogenesis, as elucidated in experimental models including subarachnoid hemorrhage and focal ischemia, may target shared pathways relevant to global cerebral injury following cardiac arrest, though validation in specific CA/CPR models remains essential.

## 4. Hyperbaric Oxygen Therapy's Protection Against Myocardial Ischemia-Reperfusion Injury and Its Neuroprotective Significance

Beyond the in-depth exploration of hyperbaric oxygen's direct neuroprotective effects, its indirect enhancement of cerebral perfusion through improved cardiac function should not be overlooked. This section will focus on elucidating this point to reveal the complete pathway of heart-brain co-protection. As the organs most sensitive to ischemia and hypoxia, the heart and brain share a core pathophysiological mechanism for reperfusion injury following resuscitation, involving common pathways such as oxidative stress, inflammatory response, and apoptosis^90^, ^91^. Research indicates that hyperbaric oxygen therapy can significantly reduce myocardial ischemia-reperfusion injury. For example, in a mouse model of myocardial ischemia-reperfusion injury, hyperbaric oxygen pretreatment reduces infarct size and improves cardiac function by activating the PI3K/Akt/Nrf2 signaling pathway and upregulating heme oxygenase-1 expression^92^.

Similarly, hyperbaric oxygen reduces inflammatory cytokine levels by inhibiting the TLR4/NF-κB pathway and modulates autophagy-related proteins such as LC3B and Beclin-1, thereby protecting cardiomyocytes^93^. These mechanisms closely resemble the anti-inflammatory and antioxidant effects of hyperbaric oxygen in cerebral ischemia-reperfusion injury, confirming the universality of the intervention strategy. More importantly, hyperbaric oxygen therapy protects the heart by stabilizing systemic blood circulation, thereby indirectly supporting cerebral perfusion and neural repair. Improved cardiac function directly enhances cardiac output and hemodynamic stability, thereby ensuring sustained and effective oxygen delivery to brain tissue following resuscitation^94^, ^95^.

## 5. Clinical study of HBO combination therapy for cerebral ischemic-hypoxic injury

The multidimensional mechanisms of hyperbaric oxygen (HBO) therapy in cerebral ischemic-hypoxic injury are becoming increasingly elucidated. Current therapeutic approaches have evolved beyond mere oxygen supplementation to encompass comprehensive strategies that leverage synergistic mechanisms. For instance, the combination of HBO with non-invasive neuromodulation techniques^96^, such as repetitive transcranial magnetic stimulation (rTMS), has demonstrated enhanced efficacy in promoting the recovery of consciousness in comatose patients compared to HBO therapy alone, underscoring the potential of combinatorial regimens^97^. This highlights a broader trend towards multimodal interventions that target distinct yet complementary pathological pathways. The successful application of combined oxygen and mechanical recanalization in other cerebral ischemic conditions further illustrates this principle. Notably, the OPENS-2 trial demonstrated that ​​normobaric hyperoxia (NBO)​​—a distinct intervention from HBO therapy, administered at normal atmospheric pressure—combined with endovascular therapy, significantly improved 90-day functional outcomes (modified Rankin Scale scores) in patients with acute ischemic stroke due to large vessel occlusion, with a favorable safety profile^98^. It is crucial to emphasize that NBO and HBO, while sharing the goal of enhancing tissue oxygenation, differ fundamentally in their pressure parameters and physiological effects, a distinction critical for accurate scientific discourse.

Combining hyperbaric oxygen (HBO) therapy with targeted temperature management (TTM), particularly mild therapeutic hypothermia, demonstrates synergistic benefits for mitigating brain injury and enhancing neurological recovery. Clinical evidence indicates that this combined approach yields superior outcomes compared to monotherapies. For instance, a clinical investigation involving patients with severe acute carbon monoxide poisoning demonstrated that combined therapeutic hypothermia and hyperbaric oxygen therapy yielded significantly better neurocognitive outcomes at the 6-month follow-up compared to hyperbaric oxygen treatment alone^99^. The complementary mechanisms underlie this efficacy: HBO directly ameliorates tissue hypoxia by elevating oxygen partial pressure, while mild hypothermia (typically maintained at 33-35°C) reduces cerebral metabolic rate, attenuates excitotoxicity, and suppresses inflammatory cascades^100^. This multi-targeted action synergistically delays the progression of secondary brain injury, positioning the combination as a promising neuroprotective strategy^101^.

Emerging evidence indicates that combining hyperbaric oxygen (HBO) therapy with acupuncture can significantly improve neurological outcomes following brain injury. A meta-analysis of 11 randomized controlled trials demonstrated that this combination was significantly superior to HBO alone in improving Glasgow Coma Scale (GCS) scores and the effectiveness rate in patients with traumatic brain injury (TBI)^102^. The neurobiological mechanisms may involve the modulation of cortical excitability and cerebral perfusion, as evidenced by functional near-infrared spectroscopy (fNIRS) studies showing that acupuncture increases oxygenated hemoglobin (HbO) concentration in the prefrontal cortex^103^. Furthermore, adjunctive therapy with certain Chinese herbal formulations has shown pro-awakening and anti-inflammatory effects in patients with severe craniocerebral injury, contributing to the overall therapeutic efficacy within an integrated treatment paradigm^104^-^106^.

## 6. Conclusions

### 6.1. Clinical Evidence and Current Challenges in HBO Therapy for Post-Cardiac Arrest Brain Injury

The current body of evidence regarding hyperbaric oxygen therapy for post-cardiac arrest hypoxic-ischemic brain injury reveals a promising yet complex therapeutic profile. Preclinical studies consistently demonstrate that HBO confers multimodal neuroprotection through distinct molecular pathways, including a 40-60% increase in superoxide dismutase activity, 35-50% reduction in pro-inflammatory cytokines such as IL-1β,and 2-3 fold upregulation of glutathione peroxidase 4, effectively mitigating oxidative stress, neuroinflammation, and ferroptosis. These mechanisms collectively contribute to the preservation of neuronal integrity and function in global cerebral ischemia models^107^. However, the clinical translation of these findings remains constrained by significant methodological heterogeneity across studies. Critical parameters such as pressure applications (ranging from 1.5 to 3.0 ATA), treatment initiation windows (2-72 hours post-ROSC), and outcome assessments lack standardization, while existing randomized controlled trials are predominantly limited by small sample sizes (the largest to date comprising approximately 118 participants)^108^. This heterogeneity underscores three fundamental knowledge gaps: the optimal therapeutic window (with animal data suggesting maximal benefit within 6 hours post-ROSC), the precise pressure-dose response relationship, and the synergistic potential of HBO with advanced cardiopulmonary resuscitation techniques.

### 6.2. Emerging Technologies and Future Therapeutic Avenues

In addressing the latter point, our research group has developed an Abdominal Lifting and Compression CPR method that demonstrates particular compatibility with subsequent HBO therapy. This technique has shown a 25% improvement in carotid blood flow, a 40% reduction in intracranial pressure fluctuations, and enhanced hemodynamic stability during compression cycles compared to standard CPR in preliminary investigations^109^-^112^.

**Figure 4:**
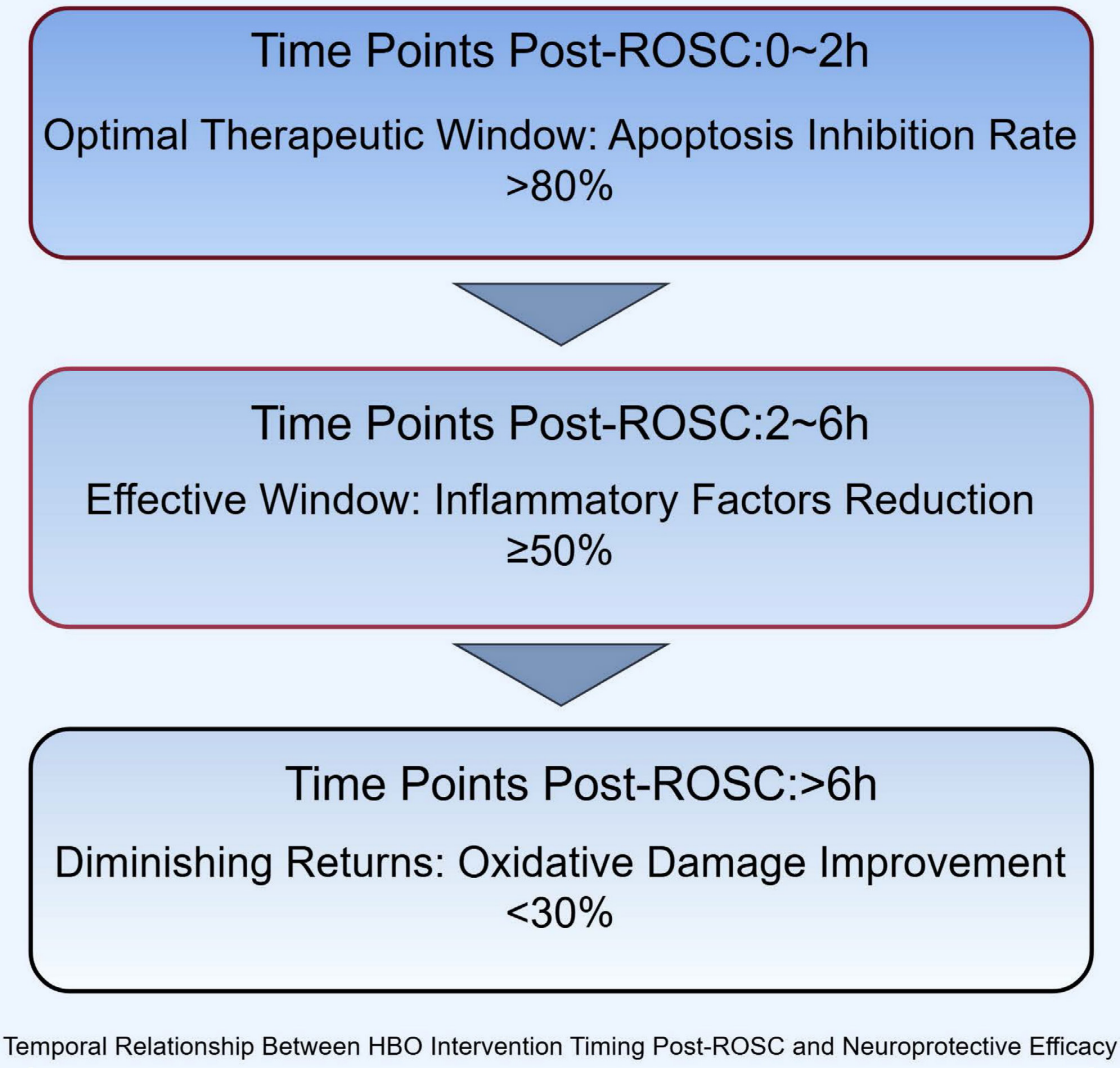
** Temporal Relationship Between HBO Intervention Timing Post-ROSC and Neuroprotective Efficacy.** Efficacy metrics: Apoptosis inhibition rate (Bcl-2/Bax), inflammatory reduction (TNF-α/IL-1β), oxidative damage improvement (MDA).

These physiological improvements may create a more favorable substrate for HBO's neuroprotective effects by optimizing cerebral perfusion prior to oxygen administration, though further validation is required. Future research priorities should focus on conducting Phase III multicenter randomized controlled trials with sufficient statistical power (recommended sample size >500), incorporating biomarker-guided therapy approaches that monitor GFAP, NSE, and S100B kinetics to personalize treatment intensity. Simultaneously, mechanistic studies exploring interactions between HBO and cellular recovery pathways, including potential synergies with mitochondrial protection and astrocyte modulation, are needed. Standardized protocol development should establish optimal pressure parameters (2.0-2.5 ATA appears favorable based on current evidence), treatment duration (60-90 minutes/session), and session frequency (daily versus alternate day) while addressing implementation challenges such as cost-effectiveness analyses and specialized training requirements for combined CPR-HBO delivery systems (Figure 4).

In conclusion, while HBO represents a promising therapeutic approach for post-cardiac arrest brain injury, its successful integration into clinical practice requires a more rigorous evidence base that specifically addresses the unique pathophysiology of global cerebral ischemia following CPR. By addressing these research priorities, we can better elucidate HBO's potential to improve neurological outcomes in this devastating condition.

## Figures and Tables

**Table 1:** Context-Dependent Regulation of HIF-1α by HBO Therapy​

Context​	Oxygen Dynamics​	Primary Effect on HIF-1α​	Key Molecular Consequences​	Functional Outcome​
Preconditioning​​ (Pre-insult)	Cyclic hyperoxia-normoxia, creating ​​controlled relative hypoxia​	Upregulation & Stabilization​	Activation of adaptive genes (e.g., EPO, VEGF); enhanced cell survival pathways^32^, ^33^	Induces ischemic tolerance; primes cellular defenses
Acute Treatment​​ (Post-insult)	Sustained hyperoxia	Downregulation & Degradation	Inhibition of HIF-1α-mediated pathological processes (e.g., excessive autophagy, inflammation)^31^, ^75^	Mitigates reperfusion injury and secondary damage

## Abbreviations

NO: Nitric oxide
ROS: Reactive oxygen species
GFAP: Glial fibrillar acidic protein
NF-κB: Nuclear factor Kappa B
HO-1: Heme-oxygenase-1
AACD-CPR: Active abdominal compression-decompression cardiopulmonary resuscitation
ATA: Atmospheres absolute
ATP: Adenosine triphosphate
BBB: Blood-brain barrier
BCL-2: B-cell lymphoma-2
BDNF: Brain-derived neurotrophic factor
BIS: Bispectral index
β-catenin: Wnt/β-catenin
CA: Cardiac arrest
CAT: Catalase
CCL2: C-C chemokine ligand 2
CIRI: Cerebral ischemia reperfusion injury
DNA: Deoxyribonucleic acid
EBI: Early brain injury
ENO: Endothelial nitric oxide synthase
EPO: Erythropoietin
GCLC: Glutamate cysteine ligase catalytic subunit
GDS: Global Deterioration Scale
GPX4: Glutathione peroxidase 4
GSH: Glutathione
HBO: Hyperbaric oxygen
HIF-1α: Hypoxia-inducible factor-1 alpha
HIBI: Hypoxic-ischemic brain injury
HMGB1: High mobility group B1
HO-1: Heme oxygenase-1
HSP70: Heat shock protein 70
IL-1β: Interleukin-1 beta
IL-6: Interleukin-6
LC3: Protein light chain 3
LPS: Lipopolysaccharide
MAPK: Mitogen-activated protein kinase
MCAO: Middle cerebral artery occlusion
MDA: Malondialdehyde
MMP-9: Matrix metalloproteinase-9
mTOR: Mammalian target of the rapamycin
mRS: Modified Rankin Scale
NMDA: N-methyl-D-aspartate
NQO-1: Quinone oxidoreductase-1
Nrf2: Nuclear factor E2-related factor 2
NBO: Normobaric oxygen therapy
NSE: Neuron-specific enolase
O₂⁻: Superoxide
O₂UCc: Oxygen consumption
ONOO⁻: Peroxynitrite
PI3K: Phosphatidylinositol 3-kinase
PFC: Prefrontal cortex
rTMS: Repetitive transcranial magnetic stimulation
RNS: Reactive nitrogen species
SAH: Subarachnoid hemorrhage
SCI: Spinal cord injury
SIRT1: Silencing regulator protein 1
SOD: Superoxide dismutase
TLR4: Toll-lik receptor 4
TNF-α: Tumor necrosis factor-alpha
TREM1: Triggering receptor expressed on myeloid cell 1
VDAC: Voltage-dependent anion-selective channel
ZO-1: Zonula occludens-1
